# Validation of Affective Sentences: Extending Beyond Basic Emotion Categories

**DOI:** 10.1007/s10936-022-09906-3

**Published:** 2022-08-11

**Authors:** Barbra Zupan, Michelle Eskritt

**Affiliations:** 1 Speech Pathology, College of Health Science, School of Health, Medical and Applied Sciences, Central Queensland University, Bruce Highway, North Rockhampton, QLD 4702 Australia; 2 Psychology, Mount Saint Vincent University, Halifax, NS Canada

**Keywords:** Sentences, Verbal content, Emotion, Validation

## Abstract

We use nonverbal and verbal emotion cues to determine how others are feeling. Most studies in vocal emotion perception do not consider the influence of verbal content, using sentences with nonsense words or words that carry no emotional meaning. These online studies aimed to validate 95 sentences with verbal content intended to convey 10 emotions. Participants were asked to select the emotion that best described the emotional meaning of the sentence. Study 1 included 436 participants and Study 2 included 193. The Simpson diversity index was applied as a measure of dispersion of responses. Across the two studies, 38 sentences were labelled as representing 10 emotion categories with a low degree of diversity in participant responses. Expanding current databases beyond basic emotion categories is important for researchers exploring the interaction between tone of voice and verbal content, and/or people’s capacity to make subtle distinctions between their own and others’ emotions.

**Supplementary Information:**

The online version contains supplementary material available at 10.1007/s10936-022-09906-3.

Our ability to identify emotions in others is closely connected to our wellbeing; contributing to academic and employment success as well as our ability to build meaningful relationships (Trampe et al., 2015). Emotion perception is a complex skill, requiring us to recognise, integrate, and interpret a number of different cues, including cues portrayed in the voice. During social interactions, we need to be able to identify the emotion conveyed through our communication partner’s tone of voice (i.e., prosody) and at the same time process the words they have used within that spoken message (i.e., verbal content). While the emotional meaning conveyed through these two sources of information may be congruent, speakers may also modify the verbal content so that it is incongruent with the prosodic cues (Pell et al., 2011; Pell & Kotz, 2011). The ability to resolve this incongruency between what is said and how it is said is essential to interpreting speech acts such as irony and sarcasm (Rockwell, 2000).

Language has been shown to influence perception and categorisation of emotion. For instance, Lindquist et al. (2006) found that speed and accuracy of facial emotion perception declined when access to emotion words and their meanings was reduced. While we know that prosody can interact with verbal content to bias how we interpret ambiguous words (Nygaard & Lunders, 2002), few studies have controlled for both emotional prosody and the semantic meaning conveyed via the verbal content to examine how these features may interact. Instead, there has been a focus on examining vocal emotion perception using sentences that include either nonsense words (e.g., “I marlipped the tovity”; Pell et al., 2011; Pell & Kotz, 2011) or words that carry no emotional meaning (e.g., “He stands on the deck”; Ben-David et al., 2011).

Though language may influence emotion perception in facial expressions (Fugate, 2013; Lindquist et al., 2006, 2014), there has been minimal exploration of the influence of language on the perception of vocal emotion expressions. Research that has included sentences with emotional verbal content did not first validate the sentences to ensure widespread consensus regarding the emotion conveyed via the verbal content (Bowers et al., 1998; Pell et al., 2009). Two studies have attempted to fill this gap by trying to identify sentences that conveyed anger, fear, happiness, sadness, or no emotion (i.e., neutral). Russ et al. (2008) presented 12 participants with a total of 130 sentences and had them use a Likert scale to rate how well the verbal content fit each emotional category. For instance, in response to the sentence, “This microwave is useless”, participants needed to rate how well the sentence fit a happy, sad, angry, or fearful context on a scale of 1 to 10, with 10 indicating a strong fit. Using a similar methodology with 48 participants, Ben-David et al. (2011) narrowed down a list to 50 sentences (10 each for happy, sad, angry, fearful, and neutral) that matched in linguistic features (e.g., number of syllables; word frequency) that could be used to investigate the interaction between emotional verbal content and prosody. The majority of these sentences did not include emotion words that matched the response options, however, there were a few examples of this (e.g., I am very angry; This is a sad moment).

Russ et al. (2008) and Ben-David et al. (2011) have provided researchers with a large set of validated sentences containing emotional verbal content, most without emotion terms, that are relatively context free. However, both lists only include sentences that convey one of four (happy, sad, angry, fearful) emotion categories, or include verbal content that is meant to be emotionally neutral. The four emotion categories are all basic emotions which don’t allow for analysis of people’s capacity to differentiate between more subtle variations (e.g., irritation, contentment). Further, the sentences only capture one positive emotion. Valence can influence our perception of vocal emotion (Pell & Kotz, 2011) but the inclusion of only one positive emotion (i.e., happy) limits this line of research because it remains unclear whether people are actually differentiating between positive and negative emotion sentences, or if they are using principles of exclusion in their responses. Further, the inclusion of only one positive response option does not allow for analysis of people’s capacity to make distinctions between positive sentences and determine if people are able to identify differences in positively-valanced messages (e.g., happy versus pride) similarly to negatively-valenced ones (e.g., angry versus sad).

The aim of the studies presented here is to generate a validated list of sentences that extend previous research using basic emotions (Ben-David et al., 2011; Russ et al., 2008) to include complex emotions. A total of 10 emotions were targeted—amusement; anxiety; compassion; contentment; disgust; interest; irritation; pride; relief; surprise. These emotions were selected to include two additional basic emotions (disgust; surprise) commonly included in emotion perception studies and tools (e.g., Kessels et al., 2014; McDonald et al., 2006), alongside a set of complex emotions that are becoming more commonly referenced in the literature (e.g., Schlegel & Scherer, 2015; Zupan & Eskritt, 2020). When combined with the lists validated by Russ et al. (2008) and Ben-David et al. (2011), the sentences validated from the current study would lead to a combined list that contains neutral, as well as a total of 14 different emotion categories; categories that include both basic and complex emotions, and a more balanced number of positive and negative emotions.

## General Method

### Participants

Participants were recruited online via both authors’ social media pages, thus a snowball sampling technique was employed. A link to the survey was also distributed through the authors’ university list serves for staff and students. Participants needed to speak English as a primary language to be included in the study.

### Stimuli

The authors and research assistants constructed a large number of emotion sentences aimed to represent 10 different emotions: amusement; anxiety; compassion; contentment; disgust; interest; irritation; pride; relief; and surprise. Due to the large number of existing validated stimuli, no sentences were constructed to target happy, sad, angry, fearful and neutral. As noted previously, a few stimuli presented by Ben-David et al. (2011) included the target emotion in the sentence, which likely influenced participants ratings regarding the degree to which the emotion response options corresponded to the sentence. Thus, in generating our stimuli, we made certain that *all* sentences were constructed to convey the target emotions without including any of the emotion words represented within the response options.

Since these studies aimed to validate sentences that crossed a broader range of emotion categories than those targeted by Russ et al. (2008) and Ben David et al. (2011), a smaller number of sentences within each category were included for each of the two studies. After independently constructing sentences, the research team first reviewed the full list to eliminate any sentences that included slang specific to culture or time (e.g., You should take a squiz; That party was lit). The research team then reviewed the remaining list to discuss the degree to which they could imagine that sentence being used in context to describe the target emotion. The aim of this process was to select a small set of sentences within each of the 10 emotion categories to ensure the total number of stimuli did not exceed 50. Selection was made on the basis of consensus; only sentences with full or majority agreement were included in the studies presented here.

### Procedure

The study was designed via the online platform Survey Monkey (www.surveymonkey.com). A copy of each survey is available in supplementary materials.^1^ The survey began with five demographic questions (i.e., gender, month and year of birth, primary language, country of residence), followed by the sentences. Sentences were presented in a fixed randomised order such that the study started and ended with a sentence aimed to convey a positively-valenced emotion, and no more than three sentences of the same valence or two sentences of the same emotion were presented in a row.

For each sentence, participants were asked to identify which emotion word BEST described the meaning of the sentence, from a randomised list of 15 different emotion words: amusement, anger, anxiety, compassion, contentment, disgust, fear, happy, interest, irritation, neutral, pride, relief, sad and surprise. This list was longer than the 10 targeted emotions because we wanted to include the traditional basic emotion labels and have a similar number of positive and negative response options. Participant ratings were based on the content of the sentence; no situational context was provided. Following completion of the fixed choice response, participants were given the opportunity to list an additional emotion word in a text box if they felt the sentence conveyed more than one emotion or conveyed an emotion that was not presented in the list of response options. Each study was expected to take approximately 15 min to complete.

### Data Analysis

The proportion of participants who identified each of the 15 possible response options for the sentences was calculated for each study. These proportions were used to identify the emotion that best represented the sentence. Though we used a consensus approach to select sentences to represent particular emotion categories, these a priori predictions were based on discussion of only a few laboratory staff members. Thus, we used the data for final identification of the emotion each sentence conveyed. Next, using the number of responses provided within each emotion category, a Simpson diversity index score was calculated for each sentence. This nonparametric statistic results in a value between 0 and 1 that indicates the degree of dispersion across categories, with values closer to 1 representing greater diversity across responses (Gregorius & Gillet, 2008; Ram et al., 2012). Thus, values closer to 0 represent more distinct representations of the specified emotion. Finally, the frequency of each emotion provided in free-text responses was calculated to identify the most frequently provided alternative for each of the sentences.

## Study 1

### Participants

Study 1 included a total of 436 participants ranging in age from 18 to 81 years (*M*_*age*_ = 43.07; *SD* = 13.19). Of these, six participants were excluded from analysis because they did not identify English as their primary language and one for not completing any of the sentence ratings, leaving 429 participants for data analysis. Of these 429 participants, 359 (84%) were female, 65 were male (15%), and 5 (1%) did not identify as either gender or preferred not to indicate. Sixty-seven per cent (*n* = 287) of participants were Australian, 26% (*n* = 111) Canadian, 5% American (*n* = 20), with the remaining 2% (*n* = 11) from five additional countries.

### Stimuli

Study 1 included a total of 45 sentences with verbal content intended to convey the following emotions: amusement (*n* = 4); anxiety (*n* = 5); compassion (*n* = 4); contentment (*n* = 3); disgust (*n* = 8); interest (*n* = 4); irritation (*n* = 4); pride (*n* = 5); relief (*n* = 4); surprise (*n* = 4). Table 1 includes the list of sentences for each emotion category. Sentences ranged from 3 to 10 words in length (*M* = 5.37) and included 3 to 11 syllables (*M* = 6.55).

**Table 1:** Simpson diversity score and mean proportion responses for top two selected emotions for each sentence for Study 1

Sentence	Simpson diversity	Emotion with highest proportion of respondents	Emotion with second highest proportion of respondents
That was priceless	.37	Amusement = .77	Happy = .17
What a funny joke!	.47	Amusement = .67	Happy = .25
Did you see that cute cat video?	.62	Amusement = .56	Happy = .18
What a clever commercial	.66	Amusement = .50	Interest = .19
That’s not fair!	.69	Anger = .46	Irritation = .27
They just kept picking on her	.80	Anger = .27	Disgust = .22
I am so tense right now	.46	Anxiety = .71	Irritation = .12
My heart is pounding	.58	Anxiety = .52	Fear = .36
I’m sorry you’re going through that	.03	Compassion = .89	Sad = .07
Oh, that poor man!	.19	Compassion = .89	Sad = .11
Please let me help you	.35	Compassion = .79	Interest = .10
She was so tender with him	.52	Compassion = .67	Neutral = .09
This is so peaceful	.47	Contentment = .70	Happy/Relief = .15
I don’t have a care in the world!	.52	Contentment = .73	Sad = .13
This is perfect	.63	Contentment = .46	Happy = .38
That is really gross	.11	Disgust = .94	Amusement/Irritation/Neutral = .01
That tastes awful	.26	Disgust = .85	Irritation = .06
What is that smell?	.66	Disgust = .42	Interest = .36
Who would do something like that?	.78	Disgust = .35	Irritation = .20
Stop! I think there’s something in there	.60	Fear = .59	Anxiety = .13
I can’t wait to hear more	.06	Interest = .90	Happy = .04
That is fascinating!	.31	Interest = .82	Amusement = .11
Why do you think that happened?	.53	Interest = .65	Surprise = .14
I like a good challenge	.64	Interest = .52	Pride = .25
You’ve asked me that a thousand times!	.13	Irritation = .93	Anger = .03
That really gets on my nerves	.20	Irritation = .89	Anger = .08
It’s driving me up the wall	.21	Irritation = .88	Anger = .07
I really wish I didn’t have to do this	.62	Irritation = .53	Anxiety = .29
How could they ignore that?	.66	Irritation = .53	Surprise = .15
Oh no, not again!	.70	Irritation = .47	Anxiety = .20
I stand up for what I believe in	.22	Pride = .87	Neutral = .05
I fight my own battles	.45	Pride = .71	Anger = .11
I finally did it	.58	Pride = .57	Relief = .29
I didn’t think I could do it, but I did	.59	Pride = .61	Relief/Surprise = .14
I feel so good about winning that	.61	Pride = .46	Happy = .41
That’s a huge weight off my mind	.07	Relief = .96	Contentment = .02
I can breathe again	.20	Relief = .89	Anxiety = .06
It’s finally over with	.26	Relief = .86	Contentment/Happy = .05
I’m so glad you’re okay	.50	Relief = .68	Compassion = .19
Wow, I never expected that	.13	Surprise = .93	Interest = .02
Who would have thought that would happen?	.25	Surprise = .86	Amusement = .09
I don’t believe it	.58	Surprise = .63	Anger = .08
Are you serious?	.74	Surprise = .42	Irritation = .21
You did not just do that!	.74	Surprise = .40	Irritation = .24
Why would someone do that?	.80	Surprise = .29	Disgust = .21

### Results and Discussion

Responses to the sentences included in Study 1 are shown in Appendix A in the supplementary materials as the proportion of participants who selected each of the response options.^2^ The emotion that received the highest proportion of responses for each sentence is shaded in grey. As shown, the sentences in Study 1 were categorised as representing 12 different emotions: amusement (*n* = 4); anger (*n* = 2); anxiety (*n* = 2), compassion (*n* = 4), contentment (*n* = 3), disgust (*n* = 4), fear (*n* = 1), interest (*n* = 4), irritation (*n* = 6), pride (*n* = 5), relief (*n* = 4), and surprise (*n* = 6). The majority of sentences (*n* = 39; 86%) were categorised according to the a priori predictions of the research team.

Table 1 lists sentences in Study 1 with their corresponding Simpson diversity index score, as well as the emotions that acquired the highest and second highest proportion of responses. While there are no specified cut-off values for Simpson diversity indices, guidelines provided in the literature indicate that values of 0.1 to 0.4 represent a low degree of diversity while values between 0.41 and 0.6 represent a moderate degree of diversity (Guajardo, 2015). Using these values, a total of 17 sentences were identified as representing an emotion with a low degree of diversity including sentences representing amusement (*n* = 1) compassion (*n* = 3), disgust (*n* = 2), interest (*n* = 2), irritation (*n* = 3), pride (*n* = 1), relief (*n* = 3), and surprise (*n* = 2). An additional 13 sentences were found to have a moderate degree of diversity including sentences representing amusement (*n* = 1), anxiety (*n* = 2), compassion (*n* = 1); contentment (*n* = 2), fear (*n* = 1), interest (*n* = 1), pride (*n* = 3), relief (*n* = 1), and surprise (*n* = 1). Overall, 30 of the 45 sentences in Study 1 showed low to moderate diversity in the emotion labels participants provided.

As per procedure, participants were able to provide an additional emotion word for any sentence they felt conveyed more than one emotion. The total number of additional words and most frequent emotions provided are listed in Table 2. For the 30 sentences selected based on their low and moderate diversity scores, the alternative responses provided via free-text generally aligned with the emotion that acquired the highest or second highest proportion of responses. For instance, for all three low diversity sentences categorised as irritation, the emotion with the second highest proportion of responses was anger; the highest proportion of alternative free-text responses was also anger.

**Table 2:** Additional responses provided via free-text response option for each sentence in Study 1

Sentence	Highest rated emotion	Total additional responses	Most frequent emotion identified (n)	Proportion of responses
That was priceless	Amusement	76	Happy (31)–Includes Gleeful (1); Joy (1)	41%
What a funny joke!	Amusement	49	Happy (22)–Includes Jovial (2)	45%
Did you see that cute cat video?	Amusement	46	Happy (19)–Includes Excited (1)	41%
What a clever commercial	Amusement	44	Interest (15)	34%
That’s not fair!	Anger	118	Irritation (47)–Includes Annoyed (3); Frustrated (5); Indignant (3)	40%
They just kept picking on her	Anger	137	Anger (33)–Includes Pissed Off (1)	24%
I am so tense right now	Anxiety	91	Fear (24)	26%
My heart is pounding	Anxiety	152	Anxiety (53)–Includes Nervous (2)	35%
I’m sorry you’re going through that	Compassion	36	Sad (14)	39%
Oh, that poor man!	Compassion	83	Sad (55)	66%
Please let me help you	Compassion	72	Interest (23)	32%
She was so tender with him	Compassion	60	Happy (12)	20%
I don’t have a care in the world!	Contentment	61	Happy (35)	57%
This is so peaceful	Contentment	79	Happy (39)	49%
This is perfect	Contentment	117	Happy (63)–Includes Excited (3); Joy (1)	54%
That is really gross!	Disgust	37	Amusement (12)	32%
That tastes awful	Disgust	29	Irritation (9)	31%
What is that smell?	Disgust	181	Interest (61)–Includes Curious (27)	34%
Who would do something like that?	Disgust	146	Irritation (22)–Includes Annoyed (1); Frustrated (1)	15%
Stop! I think there’s something in there	Fear	96	Anxiety (35)–Includes Worry (2)	36%
I can’t wait to hear more	Interest	26	Happy (9)–Includes Excited (8)	35%
That is fascinating!	Interest	47	Surprise (14)	30%
Why do you think that happened?	Interest	72	Interest (24)–Includes Curious (15); Intrigued (1)	33%
I like a good challenge	Interest	53	Happy (12)–Includes Buoyed (1); Excited (6)	23%
You’ve asked me that a thousand times!	Irritation	65	Anger (30)–Includes Contempt (1)	46%
That really gets on my nerves	Irritation	64	Anger (37)–Includes Pissed Off (1)	58%
It’s driving me up the wall	Irritation	131	Anger (54)	41%
I really wish I didn’t have to do this	Irritation	175	Anxiety (51)–Includes Apprehension (1)	29%
How could they ignore that?	Irritation	140	Anger (32)	23%
Oh no, not again!	Irritation	132	Irritation (43)–includes Annoyed (4); Dismay (1); Frustrated (12)	33%
I stand up for what I believe in	Pride	52	Determined (12)–Includes Conviction (1); Resolved (1); Steadfast (1)	23%
I fight my own battles	Pride	98	Pride (18) and Irritation (18–Includes Annoyed (1))	18% each
I didn’t think I could do it, but I did	Pride	127	Relief (38)	30%
I finally did it	Pride	171	Relief (55)	32%
I feel so good about winning that	Pride	116	Happy (39)–Includes Elated (1); Excited (3)	34%
That’s a huge weight off my mind	Relief	58	Happy (29)	50%
I can breathe again	Relief	38	Happy (17)	45%
It’s finally over with	Relief	42	Happy (15)–Includes Elated (2); Excited (1)	36%
I’m so glad you’re okay	Relief	176	Compassion (81)–Includes Caring (1); Concern (8); Empathy (6); Sympathy (2)	46%
Wow, I never expected that!	Surprise	60	Amusement (15) and Happy (15–Includes Excited (1); Joy (1))	25% each
Who would have thought that would happen?	Surprise	107	Amusement (44)	41%
I don’t believe it	Surprise	65	Disbelief (12)–Includes Astonished (1); Shock (2)	18%
Are you serious?	Surprise	120	Irritation (24)–Includes Annoyed (2); Frustration (1);	20%
You did not just do that!	Surprise	102	Irritation (24)–Includes Annoyed (1); Frustrated (2)	23%
Why would someone do that?	Surprise	178	Irritation (31)–Includes Annoyed (1); Frustrated (2)	17%

## Study 2

Study 2 was conducted to extend the number of sentences with low diversity within each of the 10 targeted emotion categories.

### Participants

Study 2 included a total of 193 participants ranging in age from 19 to 77 years (*M*_*age*_ = 40.49; *SD* = 14.46). Of these, one participant was excluded from analysis because they did not identify English as their primary language, leaving 192 participants for current data analysis. Of these 192 participants, 160 (83%) were female, 30 were male (15%), and 2 (1%) did not identify as either gender or preferred not to indicate, resulting in a nearly identical participant profile as Study 1. Sixty-two per cent (*n* = 120) of participants were Australian, 24% (*n* = 46) Canadian, 8% from the United Kingdom, with the remaining 4% (*n* = 9) from five additional countries.

### Stimuli

Study 2 included a total of 50 sentences with verbal content intended to convey the following emotions: amusement (*n* = 4); anxiety (*n* = 6); compassion (*n* = 4); contentment (*n* = 6); disgust (*n* = 6); interest (*n* = 5); irritation (*n* = 6); pride (*n* = 5); relief (*n* = 4); and surprise (*n* = 4). Table 3 includes the list of sentences for each emotion category. Sentences ranged from 3 to 10 words in length (*M* = 5.08) and included 4 to 12 syllables (*M* = 6.46).

**Table 3:** Simpson diversity score and mean proportion responses for top two selected emotions for each sentence for Study 2

Sentence	Simpson diversity	Emotion with highest proportion of respondents	Emotion with second highest proportion of respondents
That dog is such a character	.27	Amusement = .85	Happy/Interest = .06
The look on your face was priceless	.34	Amusement = .81	Happy = .10
That was entertaining	.57	Amusement = .61	Happy = .21
If you do that again, I’m going to lose it	.49	Anger = .64	Irritation = .32
This is so nerve wracking	.35	Anxiety = .79	Fear = .13
My palms are all sweaty	.38	Anxiety = .78	Fear = .11
I’m feeling jumpy today	.39	Anxiety = .77	Fear = .08
But what if they don’t like me?	.41	Anxiety = .74	Fear = .22
I can’t stop thinking about it	.62	Anxiety = .58	Interest = .15
We need to talk	.83	Anxiety = .32	Neutral = .15
I’m so sorry for your loss	.31	Compassion = .81	Sad = .18
How are you holding up?	.40	Compassion = .75	Interest = .20
Are you okay?	.45	Compassion = .69	Interest = .27
I don’t like seeing you so down	.50	Compassion = .64	Sad = .30
It’s good to see you’re okay	.58	Compassion = .52	Relief = .39
I’m so relaxed	.33	Contentment = .81	Happy = .10
I feel so comfortable here	.33	Contentment = .81	Happy = .11
This is paradise	.50	Contentment = .61	Happy = .37
This is the life	.52	Contentment = .64	Happy = .25
It’s such a pretty place	.61	Contentment = .46	Happy = .42
That smells horrible	.11	Disgust = .94	Irritation = .03
I’m not eating that	.25	Disgust = .87	Neutral = .06
I’m not touching that	.37	Disgust = .78	Fear = .11
That looks hideous	.37	Disgust = .78	Amusement = .08
That news turned my stomach	.59	Disgust = .61	Fear = .13
I couldn’t have asked for anything better	.65	Happy = .46	Contentment = .35
You got the part	.73	Happy = .44	Pride = .21
What have you got there?	.19	Interest = .90	Surprise = .08
What are you doing this weekend?	.32	Interest = .81	Neutral = .09
Tell me everything	.36	Interest = .79	Compassion = .13
How was the drive?	.38	Interest = .77	Neutral = .17
Guess what I just heard?	.59	Interest = .60	Amusement = .16
I’m so sick of this commercial	.17	Irritation = .91	Disgust = .05
My patience is wearing thin	.24	Irritation = .86	Anger = .13
I’m so over this	.40	Irritation = .77	Anger = .08
That referee needs his eyes checked	.53	Irritation = .58	Anger = .37
Leave me alone	.61	Irritation = .56	Anger = .20
I can’t believe those people	.73	Irritation = .43	Anger = .20
Look at how well you did	.45	Pride = .73	Happy = .13
I think I did very well, myself	.46	Pride = .72	Contentment = .12
You handled that really well	.60	Pride = .62	Compassion = .09
You’re so good at that	.64	Pride = .55	Happy = .18
I’m so glad that’s over	.16	Relief = .92	Happy = .03
That was easier than I expected	.58	Relief = .55	Surprise = .34
Don’t worry me like that again	.76	Relief = .38	Anxiety = .22
I never saw that coming	.20	Surprise = .89	Sad = .03
Fancy seeing you here	.45	Surprise = .72	Amusement/Interest = .08
I can’t believe I just saw that	.49	Surprise = .70	Amusement = .11
Where did that come from?	.53	Surprise = .64	Interest = .24
How did you do that so quickly?	.55	Surprise = .59	Interest = .31

### Results and Discussion

Appendix B in supplementary materials shows responses to the sentences included in Study 2 according to the proportion of participants who selected each of the possible response options; the emotion with the highest proportion of responses is shaded in grey.^3^ The results of Study 2 yielded categorisation of sentences into 12 different emotions: amusement (*n* = 3); anger (*n* = 1); anxiety (*n* = 6), compassion (*n* = 5), contentment (*n* = 5), disgust (*n* = 5), happy (*n* = 2), interest (*n* = 5), irritation (*n* = 6), pride (*n* = 4), relief (*n* = 3), and surprise (*n* = 5). Once again, the results supported our a priori predictions for the majority of sentences (*n* = 44; 88%).

The Simpson diversity index for each sentence in Study 2 is provided in Table 3 alongside the emotions that acquired the highest and second highest proportion of responses from participants. The same values as Study 1 were used to identify sentences with low and moderate degrees of diversity (Guajardo, 2015). Results of Study 2 yielded 22 sentences as representing an emotion with a low degree of diversity. These sentences crossed 9 emotion categories including amusement (*n* = 2), anxiety (*n* = 3), compassion (*n* = 2), contentment (*n* = 2), disgust (*n* = 4), interest (*n* = 4), irritation (*n* = 3), relief (*n* = 1), and surprise (*n* = 1). The 19 sentences found to have a moderate degree of diversity included sentences in 11 emotion categories including amusement (*n* = 1), angry (*n* = 1), anxiety (*n* = 1), compassion (*n* = 3); contentment (*n* = 2), disgust (*n* = 1), interest (*n* = 1), irritation (*n* = 1), pride (*n* = 3), relief (*n* = 1), and surprise (*n* = 4). Overall, in Study 2, 41 of 50 sentences were found to be labelled as representing a distinct emotion with a low to moderate degree of diversity.

The additional emotional words participants provided in the optional free-text response box for the sentences in Study 2 are listed in Table 4. The alternative responses provided for 33 of the 41 sentences identified as having low to moderate diversity, aligned with the emotion word that acquired either the highest or second highest proportion of responses.

**Table 4:** Additional responses provided via free-text response option for each sentence in Study 2

Sentence	Highest rated emotion	Total additional responses	Most frequent emotion identified (n)	Proportion of responses
That dog is such a character	Amusement	23	Happy (7)	30%
The look on your face was priceless	Amusement	24	Happy (7)	29%
That was entertaining	Amusement	28	Happy (10)	36%
If you do that again, I’m going to lose it	Anger	51	Irritation (31)–Includes Annoyed (1); Frustrated (3)	61%
This is so nerve wracking	Anxiety	24	Fear (10)	42%
My palms are all sweaty	Anxiety	33	Fear (18)	54%
I’m feeling jumpy today	Anxiety	19	Irritation (7)	37%
But what if they don’t like me?	Anxiety	35	Fear (21)	60%
I can’t stop thinking about it	Anxiety	39	Fear (6) and Interest (6–Includes Curious (1))	15% each
We need to talk	Anxiety	37	Fear (7)	19%
I’m so sorry for your loss	Compassion	29	Sad (26)	90%
How are you holding up?	Compassion	41	Interest (24)	58%
Are you okay?	Compassion	42	Compassion (21)–Includes Concern (7)	50%
I don’t like seeing you so down	Compassion	29	Sad (13)	45%
It’s good to see you’re okay	Compassion	28	Relief (12)	43%
I’m so relaxed	Contentment	23	Happy (12)	52%
I feel so comfortable here	Contentment	33	Happy (18)	54%
This is the life	Contentment	63	Happy (36)	57%
This is paradise	Contentment	37	Happy (19)	51%
It’s such a pretty place	Contentment	26	Contentment (7) and Happy (7)	27% each
That smells horrible	Disgust	11	Irritation (6)	54%
I’m not eating that	Disgust	11	Irritation (3)	27%
I’m not touching that	Disgust	45	Fear (17)	38%
That looks hideous	Disgust	23	Amusement (6)	26%
That news turned my stomach	Disgust	32	Anxiety (8)	25%
I couldn’t have asked for anything better	Happy	31	Happy (15)	48%
You got the part	Happy	40	Happy (14)–Includes Excited (2)	35%
What have you got there?	Interest	11	Surprise (3) and Interest (3–Includes Curious (1))	27% each
What are you doing this weekend?	Interest	11	Neutral (4)	36%
Tell me everything	Interest	20	Compassion (9)	45%
How was the drive?	Interest	10	Interest (3)	30%
Guess what I just heard?	Interest	18	Surprise (8)	44%
I’m so sick of this commercial	Irritation	12	Irritation (5)–Includes Annoyed (2)	42%
My patience is wearing thin	Irritation	25	Angry (17)	68%
I’m so over this	Irritation	40	Irritation (12)–Includes Frustrated (4); Annoyed (3)	30%
That referee needs his eyes checked	Irritation	27	Irritation (10)–Includes Annoyed (1)	37%
Leave me alone	Irritation	53	Angry (23)	43%
I can’t believe those people	Irritation	29	Amusement (9)	31%
Look at how well you did	Pride	27	Happy (9)	33%
I think I did very well, myself	Pride	25	Happy (9)	36%
You handled that really well	Pride	28	Compassion (7) and Relief (7)	25% each
You’re so good at that	Pride	95	Happy (27)–Includes Enthusiastic (1)	27%
I’m so glad that’s over	Relief	8	Contentment (3)	37%
That was easier than I expected	Relief	46	Surprise (17)	37%
Don’t worry me like that again	Relief	32	Fear (7) and Relief (7)	22% each
I never saw that coming	Surprise	14	Surprise (3)	21%
Fancy seeing you here	Surprise	34	Happy (11)–Includes Excited (1)	32%
I can’t believe I just saw that	Surprise	38	Surprise (12)	31%
Where did that come from?	Surprise	40	Surprise (12)	30%
How did you do that so quickly?	Surprise	28	Surprise (9)	32%

## General Discussion

The primary aim of this study was to validate a list of sentences that would extend current literature (Ben-David et al., 2011; Russ et al., 2008) to include 10 additional emotion categories. Previous studies have focused on sentences conveying only four emotions (i.e., angry, fearful, happy, sad) alongside semantically neutral sentences. The inclusion of a broader range of emotions, including seven additional positively-valenced emotions in the current study, significantly contributes to literature by attempting to validate sentences that include meaningful verbal content and convey more complex emotions. Although the number of sentences tested was not as expansive as lists validated by Ben-David et al. (2011) and Russ et al. (2008), the sentences were assessed by a significantly larger group of participants from a number of different Western countries, thus results are robust. Moreover, participants had a larger number of response options to choose from reducing the potential for inflated agreement due to chance and increasing the potential for varied interpretations and responses. Our use of diversity ratings to identify sentences that more discretely represented emotions is a strength of this study because it accounts for variations that may have been present in participants from different Western countries. As shown in Table 5, a total of 38 sentences were labelled with a low degree of diversity across the 10 emotion categories targeted in this study. The number of sentences within each category varied, ranging from only one sentence identified as representing pride, to six sentences for irritation.

**Table 5:** List of selected (Low Diversity) sentences for use in prosody studies

Emotion Category	Sentences	Study (Degree of diversity)
Amusement	That dog is such a character	Study 2 (0.27)
	That look on your face was priceless	Study 2 (0.34)
	That was priceless	Study 1 (0.37)
Anxiety	This is so nerve wracking	Study 2 (0.35)
	My palms are all sweaty	Study 2 (0.38)
	I’m feeling jumpy today	Study 2 (0.39)
Compassion	I’m sorry you’re going through that	Study 1 (0.03)
	Oh, that poor man!	Study 1 (0.19)
	I’m so sorry for your loss	Study 2 (0.31)
	Please let me help you	Study 1 (0.35)
Contentment	I’m so relaxed	Study 2 (0.33)
	I feel so comfortable here	Study 2 (0.33)
Disgust	That is really gross	Study 1 (0.11)
	That smells horrible	Study 2 (0.11)
	I’m not eating that	Study 2 (0.25)
	That tastes awful	Study 1 (0.26)
	I’m not touching that	Study 2 (0.37)
	That looks hideous	Study 2 (0.37)
Interest	I can’t wait to hear more	Study 1 (0.06)
	What have you got there?	Study 2 (0.19)
	That is fascinating	Study 1 (0.31)
	What are you doing this weekend?	Study 2 (0.32)
	Tell me everything	Study 2 (0.36)
	How was the drive?	Study 2 (0.38)
Irritation	You’ve asked me that a thousand times	Study 1 (0.13)
	I’m so sick of this commercial	Study 2 (0.17)
	That really gets on my nerves	Study 1 (0.20)
	It’s driving me up the wall	Study 1 (0.21)
	My patience is wearing thin	Study 2 (0.24)
	I’m so over this	Study 2 (0.40)
Pride	I stand up for what I believe in	Study 1 (0.22)
Relief	That’s a huge weight off my mind	Study 1 (0.07)
	I’m so glad that’s over	Study 2 (0.16)
	I can breathe again	Study 1 (0.20)
	It’s finally over with	Study 1 (0.26)
Surprise	Wow, I never expected that	Study 1 (0.13)
	I never saw that coming	Study 2 (0.20)
	Who would have thought that would happen?	Study 1 (0.25)

Across the two studies, only six sentences were labelled as representing one of the five emotion categories included in Ben-David et al. (2011) and Russ et al. (2008), despite the fact all five terms were included as potential response options. None of these achieved a low diversity score, but two were labelled were with a moderate degree of diversity—one sentence in Study 1 was labelled as fear (*Stop! I think there’s something in there*) with a diversity score of 0.60, and one sentence in Study 2 was labelled as angry (*If you do that again, I’m going to lose it*) with a diversity score of 0.49. It appears that our conceptual knowledge of emotion may be better represented by a broader set of words that capture the complexity and social aspects of language used during human interactions (Turkstra et al., 2017). However, it should be noted that we did not generate sentences to specifically target the four emotion categories previously studied, nor did we include stimuli from Ben-David et al. (2011) or Russ et al. (2008). This decision was made to maximise the number of stimuli targeting complex emotions while still ensuring a reasonable number of total stimuli for participants, but it does limit the conclusions we can draw regarding people’s conceptual knowledge of emotion. To address this, future iterations of this work should include sentences previously validated by Ben-David et al. (2011) and Russ et al. (2008) as representing happy, sad, angry, fearful, and neutral to explore whether any of these sentences would be better represented by the broader, more complex emotion categories included in the current study. Comparison of those sentences to the ones included in the current study suggest that this might be the case. For instance, a number of the sentences listed as representing happiness in Ben-David et al.’s (2011) list may have been labelled as pride if the option were available (e.g., ‘*I won an award’*; ‘*Good job, the crowd loved you’*), or even contentment (e.g., ‘*It’s a beautiful day outside*’; ‘*I really love nature’*). Similarly, sentences in Russ et al. (2008) reported to represent angry may be alternatively labelled as irritation (e.g., ‘*Why are you always testing my patience*?’; ‘*That noise is getting really annoying’*) or even disgust (e.g., ‘*He always acts like he’s better than everyone’*; ‘*Do you know how unjust that is?*’). It is also possible that sentences constructed to convey neutral may be otherwise categorised if given response emotions that represent less intense emotions. For example, in Russ et al. (2008), participants categorised the sentence, ‘*I wonder what that is about*’ as neutral. However, based on similarly constructed sentences used in the studies presented here, this sentence might be identified as interest or even anxiety if these options were available. Further exploring the influence of response options on categorisation of emotion sentences would provide further insight into our conceptual knowledge of emotion.

Although the current study included a large number of potential response options, a number of sentences were distinctly identified as representing a single emotion as indicated by low Simpson diversity scores. Sentences conveying disgust and interest appeared to be more readily categorised and easier to achieve consensus across participants, with the majority of sentences in these categories achieving low diversity scores. For instance, of the nine sentences labelled as disgust, six of these were identified as with a low degree of diversity across participant responses. Similarly, of the eight sentences identified as interest, six of these achieved low diversity scores.

It is possible that the six low diversity disgust sentences were more readily identified because they conveyed examples of physical disgust (e.g., spoiled food) which are more easily distinguishable than examples of moral disgust. Sentences we had anticipated would be labelled as (moral) disgust based on violation of social or moral norms (Chapman & Anderson, 2012) had a diverse array of responses including disgust, anger, and irritation (e.g., ‘*They just kept picking on her’*; ‘*Who would do something like that*?’). Research has shown that people often use disgust and anger interchangeably when describing social-moral norm violations (Gutierrez et al., 2011). Thus, response patterns for these sentences at least partially support the explanation that the content conveyed norm violations. Gutierrez et al. (2011) suggest that distinguishing between disgust and anger in response to social-moral norm violations is contingent on the type of moral violation. Since our sentences were presented without situational context, participants would not have had the information required to make this distinction which may explain the large variability in response to these sentences.

Interest is said to be generated in contexts where people are prompted to pay attention and/or want to further explore a situation or experience (Fredrickson, 2000, 2004). This description applies to all six of the sentences identified as interest with low diversity. Three of these sentences included the use of question words (e.g., how, what) and the remaining three used statements of attentiveness (e.g., ‘*I can’t wait to hear more*’). While interest was included in the current study as one of the seven positively-valenced emotions, alternative responses to sentences identified as Interest suggest that this emotion category does not have a clear valence. These results support our previous findings in an emotion elicitation study in which there were similar positive and negative valence ratings in response to film clips targeting Interest (Zupan & Eskritt, 2020).

Sentences identified as pride and contentment tended to garner greater diversity in responses overall; only one pride sentence and two contentment sentences achieved a low diversity score. Pride is typically classified as a positive emotion and is said to occur in situations where someone has surpassed a standard or expectation, which leads to increased feelings of self-worth (Lewis, 2008). However, some may consider expressions of pride as negative since these expressions can be perceived as self-serving and aimed to elevate one’s status at the cost of others (Oveis et al., 2010). Evaluation of responses to all sentences identified as pride seemed to support this complexity in interpretation since the emotion that received the second highest proportion of responses varied widely and included both positive and negative emotion labels (see Appendices 1 and 2 for response patterns^4^). It also possible that the characteristics or our participant sample contributed to this result. More than 95% participants in the current studies were from Australia, Canada, the United States, or United Kingdom. People from individualist, westernized cultures are less likely to associate pride with scenarios that describe others’ successes (Neumann et al., 2009). Although the sentences we constructed to target pride tended to begin with ‘I’, participants were still essentially interpreting emotion in response to a description about something external to themselves. This may at least partially explain the limited success we had in identifying sentences that represented pride with low diversity.

The response pattern for sentences labelled as contentment was more consistent than for pride, with happy found to have the second highest proportion of responses for seven of the eight sentences labelled as contentment. The emotion word most frequently listed in the free-text responses for all eight of these sentences was also happy. Although these results suggest that happy and contentment may not be well differentiated from one another, 70% of participants indicated that their preferred interpretation of the sentence was contentment for four of the eight sentences. This proportion of consensus far exceeds what would be expected by chance, even if one considers only the two response options (happy, contentment). Across the two studies, only two sentences were labelled as happy. Together, these results highlight the importance of including more than one positive response option in emotion studies since happy was not a preferred response option.

The results of this study add to the field by extending the list of potential sentences researchers can use with English speakers when examining recognition of emotion across different modalities. These sentences may be particularly useful for research investigating the complex interaction between emotional prosody and verbal content. This complex interaction has received little attention in emotion research despite the fact people manipulate the congruency between these cues to convey intention and meaning during social interactions. The lack of attention to this area of emotion recognition and inference may be due in part to the challenge of constructing sentences that do not include emotion words but are still capable of conveying a distinct emotion when presented with no context. The availability of validated sentences that extend beyond semantically neutral content and content that conveys only four basic emotions should contribute to furthering research in this area.

While we have identified 38 sentences that appear to distinctly represent 10 different emotion categories, the studies presented here have some limitations that need to be identified. First, participants were recruited via the research team’s social media pages and university list serves so the sample may not be a representative one. In addition, since the studies focused on the interpretation of language-based stimuli, we should have included education as a potential confounding variable. Finally, while we asked participants to identify the country they were from, we didn’t ask them to identify their cultural background. Although all participants reported English as their primary language and more than 95% were from Australia, Canada, United States, or United Kingdom, their cultural background may have been quite varied. However, the perception of emotion has been shown to accommodate mainstream culture for people using English in their everyday interactions after only a few years of living in an English-speaking country (Liu et al., 2017). Given that our speakers needed to identify English as their primary language to participate in this study, cultural influences in responses were likely minimal. Still, future studies should examine cultural influences on the interpretation of complex emotion sentences more carefully. This will be particularly important for researchers using these sentences to explore multisensory emotion perception, including the interaction between emotional prosody and verbal content, because cultural differences have been reported to influence the weight people place on different cues when identifying the emotion portrayed (Liu et al., 2015; Tanaka et al., 2010).

Although not a limitation per se, the number of sentences identified to represent each of the targeted emotion categories was limited. Future validation research aimed to increase the number of stimuli across these 10 categories, and particularly those categories for which we identified only a few distinct stimuli (i.e., amusement, anxiety, contentment, pride, surprise), would be beneficial. Future research might also consider validating sentences that target additional basic (e.g., contempt, joy), or complex, social emotions (e.g., shame, guilt). The role of context in the interpretation of these emotion sentences should also be explored. The words used as response options provide inherent context and constraint in perception (Feldman Barrett & Kensinger, 2010). Further validating these sentences, and those identified by Russ et al. (2008) and Ben-David et al. (2011), using different response options and formats would provide additional insight into the consistency in which these sentences represent their specified emotion category.

The sentences presented here were validated in isolation. In addition to exploring the influence of different response options, future research might explore how situational context provided via an introductory sentence or vignette may influence participants’ categorisation of the meaning the sentence conveys. Though still quite different to way in which we experience and talk about emotion in everyday life, it would provide us some initial insight into how context influences emotion perception and whether or not people are able to make use of language-based social and/or emotional cues. The majority of studies that have considered the influence of context on emotion perception have focused on the interpretation of facial emotion expressions in the presence of congruent/incongruent words (Brooks et al., 2017; Fugate, 2013; Gendron et al., 2012), vocal emotion expressions (Pell et al., 2011; Pell & Kotz, 2011), or evaluative statements (e.g., He thinks you are competent) (Schwarz et al., 2013). These studies all report an influence of context on participant responses. Further evaluating sentences such as these in context is valuable given society’s expanding use of text-based communication. Embedding these sentences within a body of text might also allow for computerized text analysis. Although traditionally used to analyse underlying intentions, emotions, and motivations in natural language samples (Tausczik & Pennebaker, 2010), computerized text analysis might be applied to bodies of text containing these sentences to provide insight into how particular patterns in word use and language structure might influence people’s perception and identification of emotion.

## Conclusion

The current study provides a set of 38 sentences that convey 10 different emotions in their verbal content with low diversity in participant responses. Importantly, these sentences were validated using English speaking adults who crossed a large age span and came from different (western) countries. This is the first study to derive validated emotion sentences for 10 complex emotions. In addition, the sentences validated in this study include a number of positively-valenced emotions which will allow researchers to further explore the influence of valence in the identification and integration of emotion cues. These results are important for providing researchers with validated sentences they can use to explore the interaction between prosody and verbal content. beyond four basic emotions and sentences neutral in verbal content. Moreover, having sentences that extend beyond four basic emotions will allow researchers to further explore multisensory emotion perception, including people’s use of emotional labels to describe their own and others’ emotions. Given that language influences perception, it is important to further our understanding of how emotion words and sentences influence our recognition and interpretation of emotion expressions, including the weight people place on this cue in multisensory emotion perception and when resolving incongruency between cues. This is particularly important for researchers working with populations known to have difficulty recognising and differentiating between emotions (e.g., traumatic brain injury) and may contribute to the development of assessment and treatment tools (Ben-David et al., 2011).

## Supplementary Information

Below is the link to the electronic supplementary material.

Supplementary file1 (DOCX 35 KB)

Supplementary file2 (DOCX 35 KB)

## Footnotes

1 Supplementary materials have been stored with Open Science Framework [Zupan, B. (2020, April 1)]. Complex Emotions Conveyed in Written Sentences. Retrieved from https://osf.io/9mwap/files/.

2 Supplementary materials have been stored with Open Science Framework [Zupan, B. (2020, April 1)]. Complex Emotions Conveyed in Written Sentences. Retrieved from https://osf.io/9mwap/files/.

3 Supplementary materials have been stored with Open Science Framework [Zupan, B. (2020, April 1)]. Complex Emotions Conveyed in Written Sentences. Retrieved from https://osf.io/9mwap/files/.

4 Supplementary materials have been stored with Open Science Framework [Zupan, B. (2020, April 1)]. Complex Emotions Conveyed in Written Sentences. Retrieved from https://osf.io/9mwap/files/.
