# Supplementary material for: Validation of Affective Sentences: Extending Beyond Basic Emotion Categories
Source: J Psycholinguist Res. 2022 Aug 11;51(6):1409–29. doi: 10.1007/s10936-022-09906-3 (PMC9646620; doi:10.1007/s10936-022-09906-3)
Supplement: Supplementary file 1 — Supplementary file1 (DOCX 35 KB) [file 10936_2022_9906_MOESM1_ESM.docx]

Appendix A. Proportion of participants to select each emotion for each sentence for Study 1.

| **Sentence** | Amuse-ment | Anger | Anxiety | Com-passion | Content-ment | Disgust | Fear | Happy | Interest | Irritat-ion | Neutral | Pride | Relief | Sad | Surprise | Simpson Diversity |
| --- | --- | --- | --- | --- | --- | --- | --- | --- | --- | --- | --- | --- | --- | --- | --- | --- |
| That was priceless. | **.77(.4)** | 0 | 0 | 0 | .02(.1) | 0 | 0 | .17(.4) | 0 | 0 | .02(.1) | .01(.1) | 0 | 0 | .02(.1) | .37 |
| What a funny joke! | **.67(.5)** | 0 | 0 | 0 | 0 | 0 | 0 | .25(.4) | .01(.1) | .02(.1) | .04(.2) | 0 | 0 | 0 | 0 | .47 |
| Did you see that cute cat video? | **.56(.5)** | 0 | 0 | 0 | .01(.1) | 0 | 0 | .18(.4) | .14(.3) | 0 | .10(.3) | 0 | 0 | 0 | 0 | .62 |
| What a clever commercial. | **.50(.5)** | 0 | 0 | 0 | .02(.1) | 0 | 0 | .05(.2) | .29(.4) | 0 | .07(.2) | 0 | 0 | 0 | .07(.2) | .66 |
| That’s not fair! | 0 | **.46(.5)** | .03(.2) | .04(.2) | 0 | .06(.2) | .01(.1) | 0 | 0 | .27(.4) | .03 (.2) | 0 | 0 | .08(.3) | 0 | .69 |
| They just kept picking on her. | 0 | **.27(.4)** | .02(.1) | .18(.4) | 0 | .22(.4) | .01(.1) | 0 | .01(.1) | .10(.3) | .02(.1) | 0 | 0 | .18(.4) | 0 | .80 |
| I am so tense right now. | 0 | .10(.3) | **.71(.4)** | 0 | 0 | 0 | .05(.2) | 0 | 0 | .12(.3) | .02(.1) | 0 | 0 | .01(.1) | 0 | .46 |
| My heart is pounding. | 0 | .01(.1) | **.52(.5)** | 0 | 0 | 0 | .36(.5) | .02(.1) | 0 | 0 | .01(.1) | 0 | .01(.1) | 0 | .05(.2) | .58 |
| I’m sorry you’re going through that. | 0 | 0 | .01(.1) | **.89(.3)** | 0 | 0 | 0 | 0 | .01(.1) | 0 | .02(.1) | 0 | 0 | .07(.2) | 0 | .03 |
| Oh, that poor man! | 0 | 0 | 0 | **.89(.3)** | 0 | 0 | 0 | 0 | 0 | 0 | .01(.1) | 0 | 0 | .11(.3) | .01(.1) | .19 |
| Please let me help you. | 0 | 0 | .02(.1) | **.79(.4)** | 0 | 0 | 0 | 0 | .10(.3) | .01(.1) | .05(.2) | .01(.1) | .01(.1) | .01(.1) | 0 | .35 |
| She was so tender with him. | .01(.0) | 0 | 0 | **.67(.5)** | .07(.3) | 0 | 0 | .06(.2) | .03(.2) | 0 | .09(.3) | .03(.2) | .01(.1) | 0 | .02(.1) | .52 |
| I don’t have a care in the world! | .01(.1) | 0 | 0 | 0 | **.73(.4)** | 0 | 0 | .12(.3) | 0 | .01(.1) | .12(.3) | .12(.3) | 0 | .13(.3) | 0 | .52 |
| This is so peaceful. | 0 | 0 | 0 | 0 | **.70(.4)** | 0 | 0 | .15(.3) | 0 | 0 | .04(.2) | 0 | .15(.4) | 0 | 0 | .47 |
| This is perfect. | .02(.1) | 0 | 0 | 0 | **.46 (.5)** | 0 | 0 | .38(.5) | .01(.1) | .01(.1) | .01(.1) | .05(.2) | .05(.2) | 0 | .01(.1) | .63 |
| That is really gross! | .01(.1) | 0 | 0 | 0 | 0 | **.94(.2)** | 0 | 0 | 0 | .01(.1) | .01(.1) | 0 | 0 | 0 | 0 | .11 |
| That tastes awful. | 0 | 0 | 0 | 0 | 0 | **.85(.3)** | 0 | 0 | 0 | .06(.2) | .03(.2) | 0 | 0 | .01(.1) | .03(.2) | .26 |
| What is that smell? | 0 | 0 | 0 | 0 | 0 | **.42(.5)** | .01(.1) | 0 | .36(.5) | .15(.3) | .04(.2) | 0 | 0 | 0 | .03(.2) | .66 |
| Who would do something like that? | .01(.1) | .15(.3) | 0 | 0 | 0 | **.35(.5)** | 0 | 0 | .06(.2) | .20(.4) | .01(.1) | 0 | 0 | .05(.2) | .16(.4) | .78 |
| Stop! I think there’s something in there. | 0 | 0 | .13(.3) | .01(.1) | 0 | 0 | **.59(.5)** | 0 | .12(.3) | .01(.1) | .03(.2) | 0 | 0 | 0 | .11(.3) | .60 |
| I can’t wait to hear more. | .03(.2) | 0 | 0 | 0 | 0 | 0 | 0 | .04(.2) | **.90(.3)** | 0 | 0 | 0 | 0 | 0 | .01(.1) | .06 |
| That is fascinating! | .11(.3) | 0 | 0 | 0 | 0 | 0 | 0 | .01(.1) | **.82(.4)** | 0 | .01(.1) | 0 | 0 | 0 | 0.05(.2) | .31 |
| Why do you think that happened? | .01(.1) | .01(.1) | .01(.1) | .02(.1) | 0 | 0 | 0 | 0 | **.65(.5)** | .05(.2) | .11(.3) | 0 | 0 | 0 | .14(.3) | .53 |
| I like a good challenge. | .06(.2) | 0 | .01(.1) | 0 | .03(.2) | 0 | 0 | .03(.2) | **.52(.5)** | 0 | .10(.3) | .25(.4) | 0 | 0 | 0 | .64 |
| You’ve asked me that a thousand times! | .01(.1) | .03(.2) | 0 | 0 | 0 | 0 | 0 | 0 | 0 | **.93(.2)** | .01(.1) | 0 | 0 | 0 | 0 | .13 |
| That really gets on my nerves. | 0 | .08(.3) | .02(.1) | 0 | 0 | .01(.1) | 0 | 0 | 0 | **.89(.3)** | .01(.1) | 0 | 0 | 0 | 0 | .20 |
| It’s driving me up the wall. | 0 | .07(.3) | .03(.2) | 0 | 0 | .01(.1) | 0 | 0 | 0 | **.88(.3)** | 0 | 0 | 0 | 0 | 0 | .21 |
| I really wish I didn’t have to do this. | 0 | 0 | .29(.4) | .01(.1) | 0 | .01(.1) | .06(.2) | 0 | 0 | **.53(.5)** | .02(.1) | 0 | 0 | .06(.2) | 0 | .62 |
| How could they ignore that? | 0 | .13(.3) | .01(.1) | 0 | 0 | .12(.3) | 0 | 0 | .02(.1) | **.53(.5)** | .01(.1) | 0 | 0 | .02(.1) | .15(.3) | .66 |
| Oh no, not again! | .02(.1) | .04 (.2) | .20(.4) | .01 (.1) | 0 | .04(.2) | .06(.2) | 0 | 0 | **.47(.5)** | .01(.1) | 0 | 0 | .10(.3) | .04(.2) | .70 |
| I stand up for what I believe in. | 0 | 0 | 0 | .03(.2) | .03(.1) | 0 | 0 | 0 | .02(.1) | .01(.1) | .05(.2) | **.87(.3)** | 0 | 0 | 0 | .22 |
| I fight my own battles. | 0 | .11(.3) | .01(.1) | .01(.1) | .03(.1) | 0 | .01(.1) | 0 | 0 | .07(.3) | .05(.2) | **.71(.4)** | 0 | 0 | 0 | .45 |
| I didn’t think I could do it, but I did. | 0 | 0 | 0 | 0 | .04(.2) | 0 | 0 | .06(.2) | 0 | 0 | .01(.1) | **.61(.5)** | .14(.3) | 0 | .14(.3) | .59 |
| I finally did it. | 0 | 0 | 0 | .01(.1) | .05(.2) | 0 | 0 | .08(.3) | 0 | 0 | 0 | **.57(.5)** | .29(.4) | 0 | 0 | .58 |
| I feel so good about winning that. | 0 | 0 | 0 | 0 | .10(.3) | 0 | 0 | .41(.5) | 0 | 0 | 0 | **.46(.5)** | .01(.1) | 0 | .01(.1) | .61 |
| That’s a huge weight off my mind. | 0 | 0 | 0 | 0 | .02(.1) | 0 | 0 | 0 | 0 | 0 | 0 | 0 | **.96(.2)** | 0 | 0 | .07 |
| I can breathe again. | 0 | 0 | .06(.2) | 0 | .03(.2) | 0 | 0 | .01(.1) | 0 | 0 | 0 | 0 | **.89(.3)** | 0 | 0 | .20 |
| It’s finally over with. | 0 | 0 | 0 | 0 | .05(.2) | 0 | 0 | .05(.2) | 0 | 0 | .01(.1) | 0 | **.86(.3)** | .02(.1) | 0 | .26 |
| I’m so glad you’re okay. | 0 | 0 | 0 | .19(.4) | .01(.1) | 0 | 0 | .07(.26) | .03(.2) | 0 | .01(.1) | 0 | **.68(.5)** | 0 | 0 | .50 |
| Wow, I never expected that! | .01(.1) | 0 | 0 | 0 | 0 | 0 | 0 | .01(.1) | .02(.1) | 0 | 0 | 0 | 0 | 0 | **.93(.2)** | .13 |
| Who would have thought that would happen? | .09(.3) | 0 | 0 | 0 | 0 | 0 | 0 | 0 | .03(.2) | 0 | .01(.1) | 0 | 0 | 0 | **.86(.3)** | .25 |
| I don’t believe it. | .04(.2) | .08(.3) | .01(.1) | 0 | 0 | .07(.2) | .02(.1) | .01(.1) | .04(.2) | .07(.2) | .04(.2) | 0 | .01(.1) | .01(.1) | **.63(.5)** | .58 |
| Are you serious? | .03(.2) | .11(.3) | .01(.1) | 0 | 0 | .02(.1) | 0 | .01(.1) | .14(.3) | .21(.4) | .04(.2) | 0 | 0 | 0 | **.42(.5)** | .74 |
| You did not just do that! | .08(.3) | .13(.3) | .01(.1) | 0 | 0 | .10(.3) | 0 | 0 | .01(.1) | .24(.4) | .02(.1) | 0 | 0 | 0 | **.40(.5)** | .74 |
| Why would someone do that? | .02(.1) | .09(.3) | 0 | .01(.1) | 0 | .21(.4) | 0 | 0 | .12(.3) | .20(.4) | .02(.1) | 0 | 0 | .04(.2) | **.29(.4)** | .80 |
