# Supplementary material for: Validation of Affective Sentences: Extending Beyond Basic Emotion Categories
Source: J Psycholinguist Res. 2022 Aug 11;51(6):1409–29. doi: 10.1007/s10936-022-09906-3 (PMC9646620; doi:10.1007/s10936-022-09906-3)
Supplement: Supplementary file 2 — Supplementary file2 (DOCX 35 KB) [file 10936_2022_9906_MOESM2_ESM.docx]

Appendix B. Proportion of participants to select each emotion for each sentence for Study 2.

| **Sentence** | Amuse-ment | Anger | Anxiety | Com-passion | Content-ment | Disgust | Fear | Happy | Interest | Irritat-ion | Neutral | Pride | Relief | Sad | Surprise | Simpson Diversity |
| --- | --- | --- | --- | --- | --- | --- | --- | --- | --- | --- | --- | --- | --- | --- | --- | --- |
| That dog is such a character. | **.85(.3)** | .01(.1) | 0 | 0 | 0 | .01(.1) | 0 | .06(.2) | .06(.2) | .01(.1) | .02(.14) | 0 | 0 | 0 | 0 | .27 |
| The look on your face was priceless. | **.81(.4)** | 0 | 0 | .02(.1) | .01(.1) | 0 | 0 | .10(.3) | .02(.1) | 0 | .02(.1) | 0 | 0 | 0 | .03(.2) | .34 |
| That was entertaining. | **.61(.5)** | 0 | 0 | 0 | .06(.2) | 0 | 0 | .21(.4) | .07(.2) | 0 | .02(.1) | .01(.1) | 0 | 0 | .03(.2) | .57 |
| If you do that again, I’m going to lose it. | .01(.1) | **.64(.5)** | .02(.1) | 0 | 0 | .01(.1) | 0 | 0 | 0 | .32(.5) | .01(.1) | 0 | 0 | 0 | 0 | .49 |
| This is so nerve wracking. | 0 | 0 | **.79(.4)** | 0 | .01(.1) | 0 | .13(.3) | .01(.1) | .01(.1) | .04(.2) | 0 | 0 | 0 | 0 | .01(.1) | .35 |
| My palms are all sweaty. | 0 | .01(.1) | **.78(.4)** | 0 | 0 | .04(.2) | .11(.3) | 0 | .01(.1) | .02(.1) | .03(.1) | 0 | .02(.1) | 0 | 0 | .38 |
| I’m feeling jumpy today. | 0 | 0 | **.77(.4)** | .01(.1) | .01(.1) | 0 | .08(.3) | .04(.2) | .01(.1) | .06(.2) | .02(.1) | 0 | 0 | .01(.1) | .01(.1) | .39 |
| But what if they don’t like me? | 0 | 0 | **.74(.4)** | .01(.1) | 0 | .01(.1) | .22(.4) | 0 | 0 | .01(.1) | .02(.1) | 0 | 0 | .01(.1) | 0 | .41 |
| I can’t stop thinking about it. | .01(.1) | .01(.1) | **.58(.5)** | .01(.1) | 0 | .01(.1) | .06(.2) | .04(.2) | .15(.3) | .04(.2) | .06(.2) | 0 | 0 | .04(.2) | .01(.1) | .62 |
| We need to talk. | .01(.1) | .11(.3) | **.32(.5)** | .05(.2) | 0 | 0 | .08(.3) | 0 | .11(.3) | .13(.3) | .15(.3) | 0 | 0 | .05(.2) | 0 | .83 |
| I’m so sorry for your loss. | 0 | 0 | 0 | **.81(.4)** | 0 | 0 | 0 | 0 | .01(.1) | 0 | .01(.1) | 0 | 0 | .18(.4) | 0 | .31 |
| How are you holding up? | 0 | 0 | 0 | **.75(.4)** | .01(.1) | 0 | .01(.1) | .01(.1) | .20(.4) | 0 | .01(.1) | 0 | .01(.1) | .02(.1) | 0 | .40 |
| Are you okay? | 0 | 0 | .01(.1) | **.69(.5)** | 0 | 0 | .01(.1) | .01(.1) | .27(.4) | 0 | .01(.1) | 0 | .01(.1) | .01(.1) | 0 | .45 |
| I don’t like seeing you so down. | 0 | 0 | .02(.1) | **.64(.5)** | .01(.1) | 0 | .01(.1) | 0 | .01(.1) | .01(.1) | .01(.1) | .01(.1) | 0 | .30(.5) | 0 | .50 |
| It’s good to see you’re okay. | 0 | 0 | 0 | **.52(.5)** | .02(.1) | 0 | 0 | .05(.2) | .01(.1) | 0 | .01(.1) | 0 | .39(.5) | 0 | 0 | .58 |
| I’m so relaxed. | 0 | 0 | 0 | 0 | **.81(.4)** | 0 | 0 | .10(.3) | 0 | 0 | .02(.1) | 0 | .07(.3) | 0 | 0 | .33 |
| I feel so comfortable here. | 0 | 0 | 0 | 0 | **.81(.4)** | .01(.1) | 0 | .11(.3) | 0 | 0 | .02(.1) | 0 | .06(.2) | 0 | .06(.2) | .33 |
| This is the life. | .01(.1) | 0 | 0 | .01(.1) | **.64(.5)** | 0 | 0 | .25(.4) | 0 | .01(.1) | .02(.1) | .02(.1) | .04(.2) | .02(.1) | 0 | .52 |
| This is paradise. | .01(.1) | 0 | 0 | 0 | **.61(.5)** | 0 | 0 | .37(.5) | 0 | 0 | 0 | .01(.1) | .01(.1) | 0 | .01(.1) | .50 |
| It’s such a pretty place. | .01(.1) | 0 | 0 | 0 | **.46(.5)** | 0 | 0 | .42(.5) | .04(.2) | 0 | .05(.2) | 0 | 0 | 0 | .02(.1) | .61 |
| That smells horrible. | .01(.1) | 0 | .01(.1) | 0 | 0 | **.94(.2)** | 0 | 0 | .01(.1) | .03(.1) | .01(.1) | 0 | 0 | 0 | .01(.1) | .11 |
| I’m not eating that. | .01(.1) | .01(.1) | 0 | 0 | 0 | **.87(.3)** | .01(.1) | 0 | 0 | .04(.2) | .06(.2) | 0 | .01(.1) | .01(.1) | 0 | .25 |
| I’m not touching that. | .01(.1) | .01(.1) | .03(.2) | 0 | 0 | **.78(.4)** | .11(.3) | 0 | .01(.1) | .04(.2) | .02(.1) | .01(.1) | 0 | 0 | 0 | .37 |
| That looks hideous. | .08(.3) | .01(.1) | .01(.1) | .01(.1) | 0 | **.78(.4)** | .01(.1) | 0 | .01(.1) | .06(.2) | .03(.2) | 0 | 0 | 0 | .01(.1) | .37 |
| That news turned my stomach. | 0 | .02(.1) | .12(.3) | .02(.1) | 0 | **.61(.5)** | .13(.3) | 0 | .01(.1) | .01(.1) | 0 | 0 | 0 | .08(.3) | .01(.1) | .59 |
| I couldn’t have asked for anything better. | 0 | 0 | 0 | .01(.1) | .35(.5) | 0 | 0 | **.46(.5)** | .01(.1) | 0 | 0 | .05(.2) | .13(.3) | 0 | .01(.1) | .65 |
| You got the part. | 0 | 0 | 0 | 0 | .01(.1) | 0 | 0 | **.44(.5)** | .02(.1) | 0 | .15(.3) | .21(.4) | .09(.3) | 0 | .07(.3) | .73 |
| What have you got there? | 0 | .01(.1) | 0 | 0 | 0 | 0 | 0 | 0 | **.90(.3)** | .01(.1) | .01(.1) | 0 | 0 | 0 | .08(.3) | .19 |
| What are you doing this weekend? | .01(.1) | 0 | .02(.1) | .01(.1) | .01(.1) | 0 | 0 | .04(.2) | **.81(.4)** | 0 | .09(.3) | .01(.1) | .01(.1) | 0 | .01(.1) | .32 |
| Tell me everything. | .03(.1) | 0 | .01(.1) | .13(.3) | 0 | 0 | 0 | .01(.1) | **.79(.4)** | 0 | .03(.2) | 0 | 0 | 0 | .02(.1) | .36 |
| How was the drive? | 0 | 0 | 0 | .02(.1) | .01(.1) | 0 | 0 | .01(.1) | **.77(.4)** | 0 | .17(.4) | 0 | .03(.1) | 0 | 0 | .38 |
| Guess what I just heard? | .16(.4) | 0 | 0 | 0 | 0 | 0 | 0 | .01(.1) | **.60(.5)** | .01(.1) | .09(.3) | 0 | 0 | 0 | .13(.3) | .59 |
| I’m so sick of this commercial. | 0 | .03(.1) | 0 | 0 | 0 | .05(.2) | 0 | 0 | 0 | **.91(.3)** | .01(.1) | 0 | 0 | .01(.1) | 0 | .17 |
| My patience is wearing thin. | 0 | .13(.3) | .01(.1) | 0 | 0 | 0 | 0 | 0 | .01(.1) | **.86(.3)** | 0 | 0 | 0 | 0 | 0 | .24 |
| I’m so over this. | .02(.1) | 0.08(.3) | .02(.1) | 0 | 0.02(.1) | .03(.3) | 0 | .02(.1) | 0 | **.77(.4)** | 0 | 0 | 0.04(.2) | .02(.1) | 0 | .40 |
| That referee needs his eyes checked. | .01(.1) | .37(.5) | 0 | 0 | 0 | .03(.1) | 0 | 0 | .01(.1) | **.58(.5)** | .02(.1) | 0 | 0 | 0 | 0 | .53 |
| Leave me alone. | .01(.1) | .20(.4) | .03(.1) | .01(.1) | 0 | 0 | .01(.1) | 0 | 0 | **.56(.5)** | .01(.1) | 0 | 0 | .19(.4) | 0 | .61 |
| I can’t believe those people. | .01(.1) | .20(.4) | .01(.1) | .01(.1) | 0 | .16(.4) | 0 | 0 | .01(.1) | **.43(.5)** | .03(.1) | 0 | 0 | .01(.1) | .15(.3) | .73 |
| Look at how well you did. | .01(.1) | 0 | 0 | .05(.2) | .01(.1) | 0 | 0 | .13(.3) | .02(.1) | 0 | .01(.1) | **.73(.4)** | .01(.1) | 0 | .03(.2) | .45 |
| I think I did very well, myself. | .02(.1) | 0 | 0 | .01(.1) | .12(.3) | 0 | 0 | .05(.2) | 0 | .01(.1) | .01(.1) | **.72(.4)** | .02(.1) | .01(.1) | .05(.2) | .46 |
| You handled that really well. | 0 | 0 | 0 | .09(.3) | .02(.1) | 0 | 0 | .08(.3) | .02(.1) | 0 | .05(.2) | **.62(.5)** | .06(.2) | 0 | .07(.2) | .60 |
| You’re so good at that. | .01(.1) | 0 | 0 | .04(.2) | .06(.2) | .01(.1) | 0 | .18(.4) | .07(.3) | .02(.1) | .05(.2) | **.55(.5)** | 0 | 0 | .02(.1) | .64 |
| I’m so glad that’s over. | 0 | 0 | .02(.1) | 0 | .02(.1) | 0 | 0 | .03(.1) | 0 | .01(.1) | .01(.1) | .01(.1) | **.92(.3)** | 0 | 0 | .16 |
| That was easier than I expected. | .01(.1) | 0 | 0 | 0 | .05(.2) | 0 | 0 | .02(.1) | 0 | 0 | .01(.1) | .03(.2) | **.55(.5)** | 0 | .34(.5) | .58 |
| Don’t worry me like that again. | 0 | .04(.2) | .22(.4) | .04(.2) | 0 | .01(.1) | .18(.4) | .01(.1) | .01(.1) | .10(.3) | .01(.1) | 0 | **.38(.5)** | .01(.1) | .01(.1) | .76 |
| I never saw that coming. | .02(.1) | 0 | .01(.1) | 0 | .01(.1) | 0 | .02(.1) | 0 | .01(.1) | .01(.1) | .02(.1) | 0 | .01(.1) | .03(.1) | **.89(.3)** | .20 |
| Fancy seeing you here. | .08(.3) | 0 | .01(.1) | 0 | .01(.1) | .01(.1) | 0 | .07(.2) | .08(.3) | .01(.1) | .03(.2) | 0 | 0 | 0 | **.72(.4)** | .45 |
| I can’t believe I just saw that. | .11(.3) | 0 | .01(.1) | .01(.1) | 0 | .07(.2) | .01(.1) | 0 | .02(.1) | .05(.2) | .04(.2) | 0 | .01(.1) | 0 | **.70(.5)** | .49 |
| Where did that come from? | .01(.1) | .01(.1) | .02(.1) | 0 | 0 | .01(.1) | .01(.1) | 0 | .24(.4) | .06(.2) | .02(.1) | 0 | 0 | 0 | **.64(.5)** | .53 |
| How did you do that so quickly? | .01(.1) | 0 | 0 | 0 | 0 | 0 | 0 | .03(.1) | .31(.5) | .01(.1) | .01(.1) | .05(.2) | 0 | 0 | **.59(.5)** | .55 |
